# Health Without Barriers: Community-Engaged Adaptation of a Whole Family-Inclusive Intensive Health Behavior and Lifestyle Program in Rural Colorado

**DOI:** 10.1007/s11121-026-01942-y

**Published:** 2026-06-26

**Authors:** Megan J. Moran, Ana Gutierrez-Colina, Virginia Jimenez, Elvia German-Palacios, Lyndsey Parrott, Talia T. Thompson, Natalia Sanchez, Addie Rzonca, Jesse Owen, Elizabeth Vargas, Caitlin Edwards, Sarah D. E. Perzow, Christine L. Chan, Matthew A. Haemer, Nicole Clark, Lauren B. Shomaker

**Affiliations:** 1 Department of Human Development and Family Studies, College of Health and Human Sciences, Colorado State University, 604 Endicott St., Fort Collins, CO 80524 USA; 2 Department of Pediatrics, Section of Endocrinology, University of Colorado Anschutz Medical Campus and Children’s Hospital Colorado, Aurora, USA; 3 Extension, La Plata County, Colorado, Office of Engagement and Extension, Colorado State University, La Plata County, Fort Collins, USA; 4 Department of Counseling Psychology, Morgridge College of Education, University of Denver, Denver, USA; 5 Department of Pediatrics, Section of Nutrition, University of Colorado Anschutz Medical Campus and Children’s Hospital Colorado, Aurora, USA

**Keywords:** Intervention adaptation, Intensive health behavior and lifestyle treatment, Rural health

## Abstract

Risk factors for type 2 diabetes (T2D) and cardiovascular diseases (CVD) are increasingly apparent in adolescence, and those in rural communities are disproportionately impacted. Intensive health behavior and lifestyle treatment (IHBLT) interventions have been shown to reduce risk factors for T2D and CVD; yet, for adolescents living in rural communities, these types of interventions are difficult to access and may be less effective due to unique barriers faced by rural adolescents. Tailoring IHBLT to the local rural context, particularly in ways that consider current and historical structural drivers of inequity, offers strong potential to address this need. Guided by the Practical, Robust Implementation and Sustainability Model with an equity focus, we co-created an adapted version of an IHBLT program, Health Without Barriers, for rural families with adolescents ages 11–19 years, emphasizing access for families living on a low-income, for adolescents whose caregivers prefer a language other than English (Spanish), and for adolescents with historically marginalized racial and ethnic identities. The aims of the current project were to (1) illustrate how participatory co-creation with community members can be systematically implemented—through an extensive initial information gathering phase coupled with iterative adaptation and additional information gathering—to make local and cultural adaptations to IHBLT, and (2) evaluate the feasibility and acceptability of those adaptations using multiple methods (self-report survey data, program attendance, and focus groups) among rural families with adolescents (*n* = 29 adolescents and *n* = 22 caregivers) who participated in the adapted IHBLT. Findings have implications for other research teams and practitioners looking to increase access and efficacy of IHBLT for the prevention and treatment of T2D and CVD in diverse and underserved rural populations.

**Supplementary Information:**

The online version contains supplementary material available at 10.1007/s11121-026-01942-y.

## Cardiometabolic Health Concerns in Adolescence

Cardiometabolic health problems, including type 2 diabetes (T2D) and cardiovascular diseases (CVD), are serious public health issues facing youth in the U.S. (Perng et al., 2024). Rising rates of T2D and CVD in adolescence are largely attributable to obesity, a major driver of preventable chronic disease, and if current trends continue, by 2050, 1 in 3 U.S. adolescents will have obesity (GBD 2021 US Obesity Forecasting Collaborators, 2024). Marked disparities exist, with higher prevalence in adolescents who identify as people of color, prefer a language other than English, live on a low income, and/or live in rural communities (Katz et al., 2021; Ogden et al., 2011; Wild et al., 2022). T2D is manifesting earlier in the lifespan and with worse severity (Lawrence et al., 2021), contributing to accelerated disease progression, earlier mortality, reduced quality of life, and substantial economic burden for families and society (Nadeau et al., 2016). Interventions to prevent or slow disease progression in adolescence are needed.

Standard-of-care for obesity involves intensive health behavior and lifestyle treatment (IHBLT), a family-inclusive, multi-component intervention (e.g., nutrition and physical activity) with at least 26 contact hours (O’Connor et al., 2024). IHBLT is effective in reducing adiposity and related cardiometabolic risk in adolescents (Savoye et al., 2014). However, access to IHBLT is limited, particularly in rural communities (Finn et al., 2024), and sustaining behavior change after the end of structured programming remains challenging (Li et al., 2025). Rural families face unique barriers, including limited availability of specialized providers and programs, long travel distances, and reduced access to safe/structured physical activity and healthy foods (Davis et al., 2024; Ko et al., 2018). Thus, even when IHBLT is available, systematic adaptation is likely necessary to support acceptability, feasibility, and long-term sustainment in rural contexts.

## Community-Engaged Co-Creation to Support Effectiveness for Rural Adolescents

Accounting for local context is critical when implementing IHBLT in rural communities. Growing evidence suggests that local adaptation improves recruitment, retention, and efficacy of evidence-based interventions for diverse populations (Thier et al., 2020). Planned adaptation, conducted in close collaboration with community members from the outset as part of a pre-implementation, information gathering phase, can support the effective translation of evidence-based interventions to rural contexts (Jolles et al., 2024). The Practical, Robust Implementation and Sustainability Model (PRISM) calls for researchers to consider perspectives and characteristics of the target population, characteristics of the external environment, and the implementation and sustainability infrastructure of a context (Feldstein & Glasgow, 2008; Fort et al., 2023). Enacting PRISM through an added layer of equity (Perez Jolles et al., 2024) includes consideration of these factors through the lens of historical and current structural drivers of inequity. This work requires participatory co-creation with a diverse set of “interest holders” (i.e., groups with an interest in the issue under discussion; Akl et al., 2024), in which they share their experience, skills, and knowledge. Approaching intervention design in this way can support alignment between the intervention and the context and, thus, lead to better outcomes in contexts with unique needs, like rural communities (Jolles et al., 2024).

## The Current Study

To prevent and treat cardiometabolic health problems in adolescence, the American Academy of Pediatrics recommends IHBLT as “early as possible” and “intensively as available” (Hampl et al., 2023). Moreover, rural families in the U.S. desire IHBLT and primary care physicians desire IHBLT as a resource for their patients (Button et al., 2025; Conroy et al., 2025). Yet these types of programs remain difficult to access and insufficiently effective for adolescents in rural communities, possibly because of a failure to account for important contextual factors when developing and delivering interventions. To address this challenge, we engaged with rural Southwest Colorado communities to co-create an adapted version of an IHBLT for area families with adolescents. Here, as with many rural regions of the U.S., disparities in cardiometabolic health outcomes exist for families living on low-income, for adolescents whose caregivers prefer a language other than English (Spanish), and for adolescents with historically marginalized racial and ethnic identities, including Hispanic and Native American (Colorado Department of Public Health & Environment, 2023). To work toward reducing disparities, we prioritized reaching these groups. The IHBLT we selected for adaptation (see Intervention Structure and Content, p. 7) was developed with interest holder input, grounded in core principles of IBHLT recommended by the U.S. Preventative Task Force (O’Connor et al., 2024). It had also demonstrated feasibility, acceptability, and efficacy with urban Hispanic families (Haemer et al., 2023), suggesting the program contained elements that appealed to one of the minoritized racial/ethnic groups in this region, making it a reasonable choice as a starting point. However, additional adaptation was needed to align the program with the rural context and the perspectives of multiple community groups, including Native American communities.

To best support key implementation outcomes, our researcher team formed a partnership with the Colorado State University Rural Extension organization. The goal of Extension is to translate scientific knowledge and extend the resources from land-grant universities directly into communities (National Institute of Food and Agriculture, n.d.), and evidence suggests that Extension may be an effective channel for disseminating T2D prevention programs, particularly in rural communities (Rafie et al., 2021). Extension specialists are well-positioned to lead efforts that maximize effectiveness of evidence-based interventions through needs assessments, tailoring, and sustainable program delivery.

In this paper, we sought to (1) illustrate how participatory co-creation with community members can be systematically implemented—through an extensive initial information gathering phase coupled with iterative adaptation and additional information gathering—to make local and cultural adaptations to IHBLT and (2) evaluate the feasibility and acceptability of those adaptations using multiple methods (self-report survey data, attendance, and focus groups) among rural families with adolescents who participated in the adapted IHBLT.

## Method

### Intervention Structure and Content

**Fig. 1:**
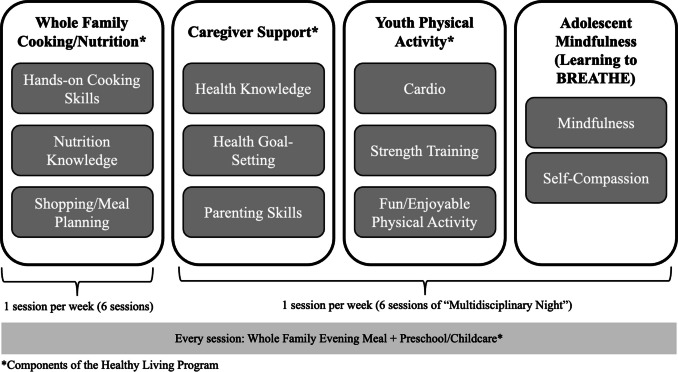
Components of the Health Without Barriers program

Health Without Barriers (HWB) integrates an IHBLT with a mindfulness training program (Fig. 1). The IHBLT, the Healthy Living Program, was developed for Hispanic children ages 2–16 years with obesity in families living on a low-income, and leads to less-than-expected BMI gain in this population (Haemer et al., 2023). The Healthy Living Program is comprised of three curricula: whole family cooking/nutrition, caregiver support, and youth physical activity (Fig. 1). The mindfulness training program is Learning to BREATHE, which was developed to support stress management and healthy coping in adolescents (Broderick, 2021). Further, pilot studies signal that Learning to BREATHE may be associated with sustained improvements in stress-related eating behavior and insulin resistance in adolescents at risk for adult obesity and/or T2D (e.g., Shomaker et al., 2019). Learning to BREATHE was originally integrated with the Healthy Living Program to create HWB in 2018–2019 by our research team, in response to feedback from focus groups with Hispanic families in urban Colorado who identified gaps in the Healthy Living Program curricula around the stress-based contributors to obesity (Haemer et al., 2023). Families participating in HWB attend 2.25-h sessions, twice a week for 6 weeks (12 sessions, 27 h). One session per week is dedicated to whole family, hands-on cooking instruction and nutrition education (*Cooking Matters User *Guide, 2023). Families receive groceries to prepare culturally attuned, healthful meals at home. During the other weekly session, families separate into groups: caregivers receive a parenting skills/goal-setting curriculum; adolescents (11–19 year-olds) receive mindfulness training and physical activity; younger school-aged siblings (5–10 year olds) receive physical activity; and toddler siblings (2–4 year olds) receive a preschool curriculum aimed at addressing fear of trying new foods. For the final ~ 30 min of both sessions, family members share a meal. All program sessions are held in the evenings at local community establishments.

### Information Gathering Phase – Qualitative Data Collection

**Fig. 2:**
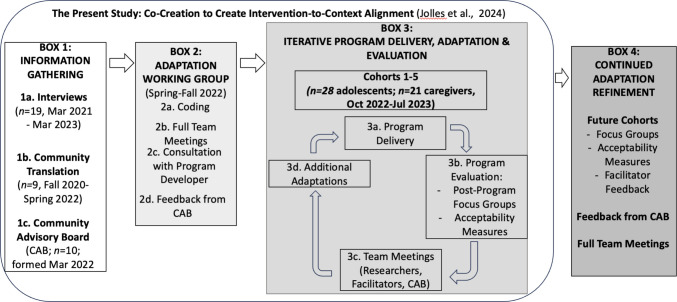
Timeline of community engagement, data collection, adaptation, program delivery, and evaluation

We engaged in an initial information gathering phase which informed a first round of adaptations (Fig. 2, Box 1). Cohort 1 received a version of the program that reflected this initial round of tailoring. We continued to collect data from program participants and program facilitators to inform the evaluation of the adaptations and make additional adaptations after each cohort. In the information gathering phase, we conducted qualitative research through three processes with three sets of interest holders: (1) interviews with local adolescents and caregivers (Fig. 2, Box 1a); (2) a Community Translation (CT; also known as Bootcamp Translation) with community members (Davis et al., 2018; Fig. 2, Box 1b), and 3) a Community Advisory Board (CAB; Fig. 2, Box 1c). Interviews and the CT were conducted simultaneously; the CAB formed after the conclusion of the CT.

#### Interviews

We conducted semi-structured interviews with adolescents and caregivers (Fig. 2, Box 1a) living in the three counties of interest (*N* = 19, 11 adolescents, 8 mothers) (Table 1). Adolescents and caregivers were interviewed separately. Participants were recruited through flyers, outreach with local youth and family-serving organizations, and referrals by CT members. Families were eligible if they had at least one adolescent between 11 and 17 years old and family history of T2D. For interviews, we focused on a family history of T2D because we have received feedback through research and community engagement efforts that this inclusion criterion is less stigmatizing than other related criteria (e.g., current elevated BMI, current T2D diagnosis), while still supporting representation of families at risk for chronic disease. Although participation was open to caregivers of any gender identity, only mothers responded with interest. Interview schedules were developed to (1) understand subjective experiences of life stressors, lifestyle behaviors, and barriers/facilitators of health behavior, and (2) get feedback on the HWB base program. Results of the first aim have been reported (Moran et al., 2024).

**Table 1:** Demographic characteristics of interview participants and program participants

	**Interviewed**			**Program**	
	Adolescent *N* = 11		Caregiver *N* = 8	Adolescent *N* = 29	Caregiver *N* = 22
Sex, *n* (%)					
Male	6 (54.5)		0	10 (34.4)	1 (4.5)
Female	5 (45.5)		8 (100.0)	17 (58.6)	21 (95.5)
Not reported	0		0	2 (6.89)	0
Ethnicity & race, *n* (%)					
Hispanic/Latino, Native American	2 (18.0)		1 (12.5)	0	0
Hispanic/Latino, White	0		0	17 (58.6)	11 (50)
Hispanic/Latino, race not reported	2 (18.0)		2 (25.0)	4 (13.8)	4 (18.2)
Non-Hispanic/Latino, Native American	3 (27.3)		3 (37.5)	0	0
Non-Hispanic/Latino, White	4 (36.4)		2 (25.0)	7 (24.1)	6 (27.3)
Non-Hispanic/Latino, race not reported	0		0	0	1 (4.5)
Neither ethnicity nor race reported	0		0	1 (3.4)	0
Preferred language, *n* (%)					
English	11 (100.0)	7 (87.5)		19 (65.5)	10 (45.5)
Spanish	0	1 (12.5)		5 (17.2)	12 (54.5)
Bilingual English/Spanish	0	0		4 (13.8)	0
Not reported				1 (3.4)	0
Highest level of education, *n* (%)					
Less than high school	Not applicable		0	Not applicable	4 (18.2)
High school diploma/GED			1 (12.5)		7 (31.8)
Post-secondary technical training			0		1 (4.5)
Some college			1 (12.5)		5 (22.7)
Associates degree			1 (12.5)		1 (4.5)
Bachelor’s degree			3 (37.5)		4 (9.5)
Some graduate school			1 (12.5)		0
Graduate degree			1 (12.5)		0
Age, *M* (*SD*), range, years	14.0 (1.5), 11–17		Not collected	14.1 (2.4), 11–19	43.2 (7.2), 30–56

#### CT

We also conducted a CT (also known as Bootcamp Translation, Westfall, 2015) (Fig. 2, Box 1b), which is a community-engaged research process to translate complex medical or research evidence into locally meaningful, culturally relevant messages, materials, and/or programs (Westfall, 2015) (for a full description, see Moran et al., 2025). CT members included *N* = 9 individuals who had lived or professional experience working with adolescents (e.g., teachers, youth afterschool program leaders), with non-profit organizations related to rural healthcare, as healthcare providers (e.g., physician, therapists), as caregivers, as community activists, or living with chronic disease. The goal of this CT—which took place between Fall 2020 and Spring 2022 and involved one full-day in-person retreat, 13 biweekly 30-min phone calls, and two half-day virtual retreats—was to gather feedback on HWB, and use this feedback as the basis for tailoring HWB. At each meeting, research team members took detailed notes. Notes were then revised for clarity and salient themes were identified. Themes were sent back to partners for member-checking and feedback. Participants were paid $250 for their time.

#### CAB

A CAB is a group of community members who provide information and ongoing support to research projects and research teams to ensure program content, delivery, and marketing is appropriate for the community (Newman et al., 2011). The CAB was formed in March 2022 and consisted of 10 individuals (see Fig. 2, Box 1c). Some CAB members had participated in the CT. Others were recruited through personal outreach by Extension staff or referrals from existing CAB members. There were no formal selection criteria; CAB members were recruited based on their involvement in the community, their interest and dedication to supporting cardiometabolic health outcomes, and/or their experience working with adolescents. Members included non-profit organization staff, healthcare providers, educators, and caregivers of children/adolescents. CAB members attended quarterly meetings to discuss program content, potential adaptations, focus groups with program participants, and implementation considerations. At each meeting, the research team took notes and reviewed them for salient recommendations, which were then incorporated into HWB adaptations.

### Program Delivery Phase – Quantitative and Qualitative Data Collection

#### Program Participants and Quantitative Assessments

Between October 2022 and February 2024, 27 families comprised of 29 adolescents (*M*_age_ = 14.1, *SD* = 2.4 years) and 22 caregivers participated in HWB (Table 1). In line with Extension’s mission to serve all people, intervention delivery was inclusive of families of all racial/ethnic identities and socioeconomic backgrounds, with at least one teenager age 11–19 years. There were no other inclusion or exclusion criteria. Notably, although recruitment included outreach to diverse racial/ethnic groups, no program participants identified as Native American (see Table 1, and Limitations). Although the original IHBLT targeted ages 2–16, inclusion of 18–19-year-olds was intentional and informed by community feedback. Families requested participation for older adolescents, and their inclusion was consistent with the program’s focus on the whole household. The enrollment approach prioritized flexibility: adolescents interested in the HWB but whose caregiver was unable to attend due to scheduling conflicts were still eligible to participate. Thus, some adolescents participated without a caregiver. Caregivers, in this case, provided consent for their child to participate. Families were recruited through partnerships with youth and family-serving organizations, school communications, healthcare provider referrals, multicultural events, community flyers, and targeted social media groups. All participants resided in two neighboring counties in Colorado. Adolescents and their caregivers could opt into assessments to characterize feasibility and acceptability of adaptations.

Ten siblings (*M*_age_ = 6.7, *SD* = 2.0 years) also attended the program, but no research measures were collected for them. Families participated over the course of five, 6-week program cohorts (Fig. 2, Box 3a). Cohort size was kept relatively small (min. 3 families, max. 10 adolescents per cohort) by design to ensure meaningful engagement and personalized support.

Adolescents and caregivers completed self-report measures of acceptability of the program overall and its components at the end of the 6-week program. These measures were adapted from the Treatment Acceptability Questionnaire (Hunsley, 1992) (see Supplementary Materials). Additionally, program staff recorded attendance, an indicator of program feasibility.

#### Post-Program Focus Groups and Facilitator Meetings

After each cohort of HWB, we held focus groups with adolescents and caregivers (Fig. 2, Box 3b). In addition, we held one focus group with program facilitators—the four individuals who delivered HWB to Cohorts 1 and 2—after Cohort 2 (May 2023). Caregiver, adolescent, and facilitator focus groups were held separately and were conducted in English and Spanish. Focus group questions were designed to elicit information about participants’ and facilitators’ experiences in the program, including questions capturing acceptability and feasibility (see Supplementary Materials). Research team members not directly involved in intervention delivery (##, ##, ##) facilitated the focus groups. Focus groups were audio recorded, transcribed and, when applicable, translated from Spanish to English. In addition to participating in a facilitator focus group, facilitators met weekly with the local Extension project program director. During these meetings, facilitators offered informal feedback on the program. The team discussed this feedback (Fig. 2, Box 3c), made decisions about whether to implement changes, and recorded changes in the master adaptation tracker.

### Data Analysis and Intervention Adaptation

#### Qualitative Data Analysis

To analyze qualitative data from both the information gathering phase (i.e., interviews, CT, and CAB), as well as the adaptation evaluation phase (i.e., focus groups), we used rapid qualitative analysis informed by the Planning for and Assessing Rigor in Rapid Qualitative Analysis framework (Kowalski et al., 2024). Rapid analysis is rigorous, pragmatic, and designed to generate actionable findings from qualitative data in a shorter timeframe than traditional approaches (Lewinski et al., 2021). Two to three members of the research team read all transcripts and meeting notes and systematically documented key points and exemplar quotes in templated summary forms. Summary forms were synthesized using analytic matrixes at each phase. Data from the information gathering phase were developed into broad, overarching themes grounded in the lived experiences and priorities of the community. Focus group data were categorized into a priori themes of program content, program structure, and program administration to provide specific guidance on program adaptation.

Our process aligns with the guidelines outlined by Rosen et al. (2018) for how to effectively use qualitative data to inform adaptations. Adaptations were made at multiple points. The largest round of modifications was made based on results of data from the interviews, CT, and the CAB prior to Cohort 1 (Fig. 2, Box 2). A working group that included researchers, Extension specialists, and community facilitators met to discuss themes derived from data analysis and to identify areas where intervention content, training processes, and delivery processes needed to be adapted (Fig. 2, Box 2b). The developers of the Healthy Living Program and Learning to BREATHE provided guidance to ensure core elements of the interventions were retained (Fig. 2, Box 2c). Key adaptations were presented to the CT and CAB for additional input (Fig. 2, Box 2 d). In addition to this initial round of adaptation, after each of the five cohorts of program delivery, more adaptations were made based on data from post-intervention focus groups with program participants (Fig. 2, Boxes 3b, 3c, 3 d).

To support understanding of when adaptations were made, what was changed, and the rationale for modifications throughout this iterative and dynamic process, we used a modified version of the Framework for Reporting Adaptations and Modifications to Evidence-based Implementation Strategies (FRAME-IS) (Miller et al., 2021) (see Supplemental Table 2 for an excerpt of the adaptation tracker). Although this study focused on adapting an intervention program, the adaptations extended beyond intervention content to include substantive changes to implementation processes; thus, the FRAME-IS was the most suitable and flexible framework. We recorded when the adaptation was made and who primarily participated in the decision to make it (research team, participants, facilitators, CT, interviewees, and/or CAB). We also recorded what was modified. We utilized the most relevant options from the FRAME-IS, including changes in packaging/materials, adding elements, removing/skipping elements, shortening, lengthening, loosening structure, refining content, or other. We also noted the specific theme from qualitative data that most closely aligned with the adaptation. We recorded the primary goal of the adaptation (increasing reach/engagement, increasing retention, improving feasibility, improving fit with participants, addressing cultural factors, improving effectiveness, reducing cost, or increasing acceptability/satisfaction). We calculated frequencies and percentages for each key FRAME-IS dimensions.

**Table 2:** Themes from information gathering phase, representative excerpts from community translation (CT) meetings notes and/or quotes from interviews, and example associated program adaptations implemented in Cohort 1

Themes	Representative excerpt from CT meeting notes or quote from interview	Example program adaptation(s)
Allow for and embrace culturally bound ideas about health	• The notion of cause and effect is a very Western concept. American Indian worldview is relational and holistic. – CT Meeting Notes • “Spirituality is very important for us because it is like a way for us to focus on being calm and have faith that what is causing us stress at that moment will be fixed, yes?”‬—Interviews, Hispanic mother‬‬	• To Session 1, added an opportunity for participants to reflect on and discuss how their culture and traditions have influenced their ideas about health
Center the rural experience	• [Program facilitator should be] Someone who has spent a good chunk of their life here. That understands how people down here think, because it is different especially if you are comparing it to a big city. – CT Meeting Notes • “And it doesn't even matter if they're like a boy or girl, but just like someone who's like funny, and that will like be there. If you wanted to like talk about your problems to them, like someone you can trust. But someone who like kind of like understands what you're going through.” – Interviews, Hispanic teen • (*In response to a question about the frequency of sessions*) Depends on how far a distance we’re “stretching.” Could work 2 times a week if it’s less than 20 min away – CT Meeting Notes	• Added to desired facilitator qualifications, “Demonstrates familiarity with the community where the program is offered,” while remaining flexible due to extremely small hiring pools in rural communities • When facilitators are being trained, they receive a list of goals for their professional development, including qualities related to cultural humility and cultural competence (e.g., Facilitator is familiar with cultural barriers and problems families face and incorporates or allows families to reflect on the following experiences and how they impact their health) • In the Physical Activity Facilitator Manual, additional guidance was added to remind the facilitator to acknowledge there are many ways and places to be physically active (e.g., physical activity can be doing chores/dancing/etc.)
Honor family autonomy and leverage the strength of the family unit	• Avoid using the word priority. The word priority seems judgmental [….] We do not want to tell people what their priorities are. – CT Meeting Notes • Empower everyone in the household. What role do they have to play, and make sure that everyone is playing a role that they are comfortable in and can thrive in. – CT Meeting Notes • “Western family is usually just your kids and your spouse. For me, it's my mom, my brother, my extended family, my sisters and my brothers that are cousins, but we don't call them cousins. We were raised, our kinship system is closer‬.” – Interviews, Native American mother	• Program recruitment materials were revised to address and be appealing to the whole family • Incorporated mindfulness into every caregiver class to increase alignment between teenager and caregiver programs • Included additional guidance for Nutrition Education facilitator to acknowledge consistent mealtimes and planned location may not always work with busy family schedules • In facilitator training, included strategies for how to communicate in ways that are autonomy-supportive and empowering • In hands-on cooking class, elicited ideas from families on recipes they would like to cook as part of the program

Analysis of qualitative data was guided by the “Big-Tent” framework for quality in qualitative research, with a focus on engaging in practices to ensure credibility (i.e., trustworthiness, believability, usefulness) and transferability of findings (Tracy, 2025). These practices include triangulation of data across multiple sources (e.g., interviews and data collected from the CT; focus groups from multiple cohorts) and member reflections in which we continuously brought results of qualitative analysis back to participants (i.e., interviewees, CT, CAB, and program participants) for their opinions, insights, and critiques. In addition to credibility, we aimed for resonance and transferability: the goal was to accurately reflect the experiences of the interest-holders we engaged with for this research and provide examples that other researchers and practitioners conducting similar work could connect with (Tracy, 2025).

#### Quantitative Data Analysis

We examined descriptive statistics to characterize acceptability and feasibility (attendance) overall and by cohort.

## Results

### Qualitative Results: Information Gathering Phase and Initial Adaptations

Across the three sources of data in the pre-implementation, information gathering phase, we generated three themes. These themes, and the specific quotes and detailed notes from which they were generated, informed an initial round of program adaptation. Themes, representative quotes/excerpts from notes, and example adaptations are summarized in Table 2.

#### Theme 1: Embrace Culturally Bound Understandings of Health

Interest holders petitioned for a culturally and contextually based understanding of what health means. It was important to interest holders that uniquely Native American and Latino notions about health and wellness be given space within the program. Interest holders emphasized that an important aspect of health is emotional or spiritual health and that joy and happiness are critical ingredients to this dimension. They voiced that the program should create opportunities for fun and entertainment, as well as family togetherness to foster and solidify positive relationships. This theme also encompasses interest holders’ assertions that Native American and Latino families have distinct ideas about what health means, relative to White families. For example, we heard that the Native American understanding of health is more relational and holistic, compared to the Western/White concept of health articulated in the HWB program, which emphasizes cause and effect. When interest holders made comments related to this theme, they also identified challenges that could come up; for example, they suggested that there are generational differences in Native American and Latino communities regarding level of comfort talking about mental health, and these phenomena would need to be accommodated within the program.

#### Theme 2: Center the Rural Experience

Interest holders said that rural life creates unique opportunities (e.g., accessible natural areas and outdoor walking paths) and barriers (e.g., long distances to gyms) to health, and they spoke about how they hoped the program would address those opportunities and barriers. Program staffing was a central focus—facilitators should be warm and energetic, but most importantly, they should identify as part of the community and understand the rural experience. There were also important aspects to consider related to program location, schedule, and seasonality given the long distances that families living in frontier regions neighboring rural or agricultural communities would potentially need to drive. Because an important component of the program is encouraging families to initiate and maintain a practice of physical activity on their own outside the weekly sessions, interest holders also noted that it would be important to recognize that families might not have access to a safe place for children to be active. This feature was noted as a unique challenge of rural living.

#### Theme 3: Honor Family Autonomy and Leverage the Strength of the Family Unit

Interest holders were enthusiastic about the whole-family approach of the HWB program, and they endorsed the idea that entire families are involved in and affected by lifestyle choices. As such, they suggested that HWB should intentionally leverage the “strength of the family” in addition to supporting individual youth. Community members and interviewed adolescents and caregivers felt strongly that autonomy of the family must be respected. Families are the ultimate authority on their own health and choices. Further, families have different needs and there is no “one-size fits all” guidance when it comes to health. Interest holders emphasized that all program content and implementation processes, including facilitators’ behavior and speech, should reflect awareness of and willingness to understand their own biases.

Table 2 illustrates example adaptations that were made based on these findings from the initial information gathering phase and their aligned theme.

### Evaluation and Iterative Adaptation

#### Quantitative Acceptability and Feasibility

Fifteen adolescents and 13 caregivers in cohorts 2–5 completed post-program acceptability surveys. Participants in cohort 1 did not complete acceptability surveys. Caregiver acceptability ratings were high, and adolescent ratings were slightly lower. In response to the question “Would you participate in the program again?” the average adolescent rating was *M* = 3.40/5.0 (*SD* = 1.45), with 47% saying “probably” or “definitely yes.” On the same question, the average caregiver rating was *M* = 4.38/5.0 (*SD* = 1.19), and 80% said “probably” or “definitely yes.” In terms of how much they enjoyed participating, adolescents reported *M* = 3.67/5.0 (*SD* = 1.05) and caregivers reported *M* = 4.15 (*SD* = 1.07); 53% of adolescents and 67% of caregivers said they “mostly” or “definitely” enjoyed participating. To the question, “Would you recommend the program to family or friends?” 53% of adolescents said “probably” or “definitely yes” (*M* = 3.80/5.0, *SD* = 1.37). In contrast, 87% of caregivers said “definitely yes” (*M* = 4.69, *SD* = 1.11).

On average, adolescent and caregiver participants attended 10/12 sessions (22.5/27 contact hours, 83.3% possible dosage; Supplemental Table 1), with considerable variability across cohorts. Median adolescent attendance ranged from as low as 7.5 sessions (16.9 contact hours) in cohorts 3 and 4, to 11 sessions (24.8 contact hours) in cohort 5. Caregiver attendance also varied by cohort, ranging 6 sessions (13.5 contact hours) in cohort 1 to 10.5 sessions (23.6 contact hours) in cohort 2. Sometimes adolescents attended without a caregiver, and vice versa.

#### Focus Groups

Rapid qualitative analysis of focus groups from Cohorts 1 to 5 revealed praise for the program, as well as constructive feedback on ways that HWB could be improved to optimize acceptability and feasibility. Table 3 summarizes adaptations and their justification based on focus group input. Qualitative analysis of focus groups indicated that later focus groups (i.e., cohorts 4/5) yielded fewer ideas for improvements relative to earlier cohorts (Supplemental Table 3) and suggestions were more individualized and less generalizable.

**Table 3:** Select adaptations to the Health Without Barriers (HWB) program and associated justifications based on focus group feedback from program participants and program facilitators

	**Adaptations**	**Justification**
**Program Content & Curriculum**	• Modified **Caregiver Education Program facilitator manual** to be more developmentally attuned to the unique challenges of parenting adolescents, as it relates to health behaviors; initiated a quarterly **Community Speaker Series** presented by volunteer content experts, outside of the program sessions, covering topics such as behavior management	• Caregivers shared that, in the Caregiver Education Program, they would like more conversations about the challenges of parenting teens
	• Modified **Learning to BREATHE (L2B) facilitator manual**, adding more examples of applications of mindfulness in interpersonal situations • The **L2B and caregiver education facilitator manuals** were restructured to indicate priority topics each week and required content was condensed	• Adolescents appreciated they were able to apply mindfulness practices to interpersonal conflict • L2B and parent education facilitators observed that the designated time was insufficient to fully cover the curriculum content
	• Incorporated **family-contributed cultural recipes** into cooking sessions	• Parents appreciated that recipes were creative and flexible, but wanted greater representation of meals from different cultures
	• Planned to add a second **Family Physical Activity** Night to the 6-week program • Modified the cardio portion of the **Physical Activity manual** to elicit activities that adolescents would like to do	• Caregivers and adolescents said Family Physical Activity Night was a highlight of the program and requested additional opportunities to interact as a whole family • Adolescents said they would like to have more of a say in the specific activities they did during Physical Activity
**Program Structure**	• Added a **“Meet and Greet” session** prior to the first session to build community and to provide a dedicated time for icebreakers and introductions outside of formal programming • Modified **program timing**, adding 15 min at the beginning of each session, during which time adolescents and siblings were encouraged to get snacks and use the restroom, and participants were able to check in with facilitators	• Caregivers and adolescents appreciated the strong community atmosphere of the program, which they attributed in part to the caring and knowledgeable facilitators and staff • Facilitators reported several challenges: adolescents visiting the restroom during the session (particularly in large groups) was disruptive; participants arrived hungry upon arriving at the program and facilitators had difficulty completing the session content due to time needed for icebreakers and introductions
**Program Administration**	• Implemented small group “**cooking teams**” and age-based cooking stations for younger siblings with developmentally appropriate meal preparation tasks	• Caregivers and facilitators expressed that cooking was hard to manage with a large group, and they wanted more adolescent and sibling involvement in cooking
	• Staff developed **operating procedures** for delivering the program bilingually (Spanish/English) • Established a **collaboration** with the Durango Education Foundation to provide organizational support for their fall and spring resource fairs—including booth mapping, QR code development for all organizations, interpretation services, and family referrals—while receiving HWB program promotion	• Caregivers, particularly in cohorts with both Spanish and English speakers, said that the interpretation skills of facilitators and staff (bilingual Spanish–English) brought the group together • Families requested one-stop access to educational, social, and health services with Spanish interpretation support, Durango Education Foundation needed organizational help, and HWB needed recruitment and community partnership opportunities
	• Expanded **snack offerings** to stave off hunger before dinner • Modified program **operating procedures** to give families the option to take dinner to-go on an as needed basis, in case they needed to leave early due to other family obligations	• Caregivers and adolescents said eating late is difficult • For some caregivers, the late end time of the program was a challenge
	• Made home practices more **accessible** by adding QR codes to home practice workbook and developed a **resource** for caregivers that gave them an overview of teens’ home practice	• Caregivers wanted more communication about adolescent at-home practice so they could help reinforce it
	• Added a question to **pre-program surveys** to gather precise information about communication preferences	• Caregivers appreciated text reminders and would like more reminders around weekly goals

### FRAME-IS Reporting of Adaptations

Throughout the process of making adaptations, we systematically recorded what was modified, when, and justification for the change in a tracker based on the FRAME-IS (Miller et al., 2021). Adaptation details are reported in Tables 2 and 3. In terms of timing, 29% of adaptations were made after the information gathering phase (Fig. 2, Box 2), prior to the start of Cohort 1. For a more detailed breakdown of the timing of adaptations by program component, see Supplemental Table 3. In terms of where the ideas for specific adaptations originated, most came from participant focus groups (36%), followed by the research team (26%), facilitators (20%), and interviews/Community Translation/CAB (18%). Adaptations largely were made to either program content (42%) or implementation procedures (45%), with a smaller number of changes to facilitator training (10%) and evaluation procedures (3%). In terms of what features specifically were modified, we revised facilitator manuals (67%), participant-facing materials (e.g., workbooks; 9%), general operating procedures for the program (10%), training procedures and materials (10%), research protocols and materials (3%), and recruitment materials (e.g., flyers; 1%). Most modifications involved adding elements (64%); other modifications included refining elements (19%), removing elements (5%), reordering modules/segments (6%), condensing content (4%), or changes to packaging/materials (2%). The most common rationale reported for adaptations was to increase acceptability/satisfaction (51%) and/or to improve effectiveness (30%). Other goals included increasing feasibility (8%), addressing cultural factors (6%), improving reach or engagement (3%), and increasing retention (2%).

## Discussion

Despite the promise of IHBLT for cardiometabolic health problems in adolescents (Li et al., 2025), these interventions are difficult to access (Finn et al., 2024), especially for adolescents who live in rural areas (Dugani et al., 2021). In line with PRISM, adapting evidence-based interventions in close collaboration with interest holders may support greater effectiveness and more equitable dissemination (Feldstein & Glasgow, 2008; Jolles et al., 2024). However, details related to the formative processes of intervention adaptation to align with the perspectives and characteristics of a target population, as well as results of the adaptation in terms of acceptability and feasibility are not often explicitly documented. Detailed documentation of adaptation processes is important for prevention science, particularly given the central tension between adaptation and fidelity (Bopp et al., 2013; Kemp, 2016). In this study, we aimed to illustrate how participatory co-creation with interest holders can be implemented to systematically adapt an IHBLT, as well as to evaluate the resulting adaptations in terms of feasibility and acceptability when delivered to rural families with adolescents. Themes from the information-gathering phase highlighted that the intervention needed to be modified to better reflect culturally-informed views on health, offer feasibility for rural families, and support and empower family autonomy. Focus groups with the families who received the adapted program led to additional, iterative refinements. The program was feasible, as indicated by average attendance of > 80%, and well-liked in many regards, but families, especially in the first few cohorts, also had suggestions to make it more relevant, practical, and enjoyable. Although Native American perspectives informed the adaptation process through information gathered through interviews, the CT, and the CAB, these perspectives were not represented in the pilot program sample and post-program focus groups. As such, the acceptability and feasibility findings cannot be assumed to generalize to Native American adolescents and families. This discrepancy between participation in the adaptation process and representation in program delivery highlights the need for future work to prioritize recruitment and implementation strategies developed in partnership with Native American communities (Blue Bird Jernigan et al., 2020).

Consistent with the PRISM framework, we sought to balance fidelity to the core “functions” or components of IHBLT (e.g., 26 + contact hours of whole-family programming that includes caregiver education, hands-on cooking/nutrition education, child and adolescent physical activity) while applying flexibility in adapting the “forms” of these elements to the local context (Feldstein & Glasgow, 2008). In addition to the numerous adaptations that were made, there were also many possible adaptations that the research-Extension team discussed but did not ultimately enact. At various points, suggestions for the program were offered that fell outside the scope of health behaviors and lifestyle modifications (e.g., caregiver requests for support with managing challenging adolescent behaviors that were not applicable to most families in the program). In these cases, we made referrals to mental health services outside the program, rather than adaptations. In complex, multi-faceted interventions, like HWB, it can be difficult to identify the core components of the intervention, or those functions that are most responsible for its efficacy on key outcomes; nevertheless, doing so is critical when conducting adaptations (Corpus-Espinosa et al., 2025). Consultations with the program developer (Fig. 1, Box 2c) ensured that essential intervention elements were preserved (Corpus-Espinosa et al., 2025); however, there were multiple points during working group meetings when the best path forward in terms of whether or not to adapt was not entirely clear. In these instances, adaptation decisions were guided by qualitative feedback across data sources and cohorts, with modifications enacted only when they enhanced contextual fit while remaining aligned with core IHBLT components (Kemp, 2016). The iterative and ongoing nature of the information gathering and adaptation process through multiple implementations of the program further supported a balance between fidelity and flexibility (Bopp et al., 2013). If the research-Extension team heard a piece of feedback in focus groups with one cohort, but were uncertain about the generalizability of the suggestion, the team waited to see if that feedback came up again during the focus group with the following cohort. Systematic documentation of adaptations and regular meetings with Extension partners minimized unilateral decision-making, fostered co-creation, and built trust in the research-community partnership. This type of planned adaptation can encourage ownership of an intervention by a local community, as well as commitment to continued implementation of the intervention with fidelity, thereby supporting sustainability (Kemp, 2016). Future studies utilizing complex experimental designs such as the Multiphase Optimization Strategy (MOST) are well suited for identifying components or adaptations for inclusion or rejection, and refinement of selected elements. More work is needed to understand how to enhance engagement, support attendance, and/or boost effects, both during program delivery and after the 6-week intervention period, through digital tools and other strategies.

Interest holders’ multiple intersecting identities shaped their perspectives on the HWB program and the ways it would need to be adapted. Aspects of identity that featured most prominently were rural location, race/ethnicity, and family identity (i.e., the sense that families have their own unique, family-level needs, priorities, and strengths). Themes derived from the CT, CAB and interviews connected in key ways to prior literature. For example, individualism, self-sufficiency, and self-reliance are commonly held values in rural communities (Coombs et al., 2022). This finding aligns with a theme from our data related to family autonomy. Interest holders rejected program elements that seemed to be “one-size-fits-all” recommendations or plans and instead endorsed a more personalized approach. Additionally, interest holders in the CT and CAB who identified as Native American most resonated with a relational and holistic conceptualization of health in the HWB program, which is consistent with the First Nations Mental Wellness Continuum Framework that emphasizes the importance of purpose, hope, and belonging to overall wellness and balance (Health Canada, 2015). Latino families appreciated the program was offered in English and Spanish, aligning with prior research on the importance of language accessibility in T2D prevention programs (Tabak et al., 2015). Interest holders’ intersecting identities shaped their perceptions of the program and feedback. Variations in identity (racial/ethnic identities but also profession, education, and socioeconomic status) likely affected the adaptations to HWB that were made based upon interest holder feedback. This work highlights the complexities of tailoring for multiple groups, and suggests a need for explicit application of intersectionality to intervention development (Ghasemi et al., 2021).

The pre-implementation, information gathering phase yielded numerous opportunities to better align the forms of the intervention functions to the local context in ways that address structural inequities (Feldstein & Glasgow, 2008; Jolles et al., 2024). For example, to address the suggestion to increase family autonomy, and to bring program procedures into greater alignment with principles of cultural humility (D. E. Davis et al., 2018), we adapted the procedures of the cooking class component to include recipes from participating families, rather than relying only on the preexisting program recipe collection. This adaptation is consistent with principles of food sovereignty, and highlights a key opportunity within HWB for supporting the “right of people to healthy and culturally appropriate food produced through ecologically sound and sustainable methods” (Blue Bird Jernigan et al., 2021). Because a key component of HWB involves the planning, purchasing, and preparation of meals, the program is well-positioned to advance food sovereignty; however, doing so in a more robust way will likely require collaboration with additional community partners integrated into the food system. This is particularly relevant given that provision of groceries is part of HWB. Funds for groceries in this project were supported by the grant; however, more sustainable models are needed in future work. The food system is an example of another layer of contextual factors (e.g., external environment and implementation/sustainability infrastructure) that PRISM prompts prevention scientists to consider (Feldstein & Glasgow, 2008). This prerogative is particularly important, considering the historical and current injustices experienced by Native American and Latino individuals related to food systems (Kitch et al., 2021). Future work might consider a dedicated adaptation process aimed at identifying opportunities for promoting food sovereignty within HWB.

Attempts to adapt IHBLT to be culturally relevant to specific populations should involve tailoring focused on facilitator characteristics (e.g., facilitators should share lived experience and identities with participants) and procedural elements (e.g., integration of communication technologies), in addition to intervention content (Wadi et al., 2022). These types of adaptations figured prominently in the co-creation process in the current project. We not only prioritized hiring facilitators from the community, we also provided trainings in cultural safety and inclusivity, specifically in the tenets of a multicultural orientation framework (Watkins et al., 2019). Providing this training is consistent with T2D prevention programs that have been adapted to increase equity (e.g., Cranston et al., 2025). Possibly because of this adaptation, which was informed by the information gathering phase and undertaken prior to cohort 1, families were highly satisfied with facilitators. Specifically, they valued the strong sense of support they felt from facilitators and appreciated access to bilingual facilitators, among other qualities. These findings suggest more research is needed on the role of the therapeutic alliance [i.e., a collaborative helping relationship characterized by respect, empathy, warmth; Sturgiss et al., 2016)], which has been cross-sectionally associated with improved outcomes in primary care obesity management, but is rarely explicitly targeted in lifestyle interventions (Sturgiss et al., 2016). Additionally, we made various procedural adaptations, including responding to families’ desire for more text message reminders about the program and “homework.” This adaptation is consistent with limited research on the use of mobile technology in family-based lifestyle programming for individuals who identify as Latino and/or Native American. In these studies, text messages were well received and viewed as a helpful way to foster accountability (e.g., Tomayko et al., 2021). However, there are equity and access considerations when using mobile technology (Richardson et al., 2022), and more work is needed to understand these barriers.

Patterns of quantitative acceptability data were consistent with prior evaluations of acceptability of HWB among adolescents and caregivers. Specifically, similar to past work conducted in an urban setting, we found that caregivers endorsed higher ratings of acceptability on all measures, relative to adolescents (Gutierrez-Colina et al., 2025). Caregivers and adolescents have distinct perceptions related to the enjoyment and perceived benefit they derive from participating in family-based programs (Biggs et al., 2023; Tobin et al., 2021), and caregivers tend to endorse higher acceptability than adolescents in full-family programs (Stern et al., 2023). A strength of this study is the use of multiple methods to assess acceptability. Follow up research should further probe quantitative acceptability ratings through targeted qualitative data collection to understand these ratings.

In making adaptations, we sought not only to address suggestions for improvement, but to amplify those aspects that families perceived were especially acceptable or effective. For example, there was enthusiasm for the whole family nature of the program. Prior research suggests participation of the whole family may be especially valuable for Native American and Latino families because of the importance of family connection and community to Indigenous mental health and wellbeing (O’Keefe et al., 2022), and the centrality of familism within Latino communities (Hernández & Bámaca-Colbert, 2016). In focus groups, participants requested more whole family activities and, accordingly, we added a second opportunity for whole family-physical activity, as well as a whole family mindful moment at the beginning of each night. Additionally, when families shared that they valued the community feel of the program, we sought to boost this quality and added a “meet and greet” gathering prior to the first session. Group cohesion is a “critical determinant” of treatment effectiveness in group psychotherapy and is associated with more regular attendance and increased engagement at group sessions (Forsyth, 2021); thus, we might anticipate that the cohesiveness of the group would be important in HWB. Creating opportunities to increase identification with the group, positive group-level affect, and group focus on a shared goal (Forsyth, 2021), such as through a pre-program orientation session, may be one way to increase group cohesion. One study of a behavioral intervention for chronic pain found that holding a group-based orientation session was associated with higher retention and adherence, relative to the intervention without the orientation session (Mayhew et al., 2020). In this study, the orientation session was primarily focused on explaining study procedures and utilizing motivational interviewing techniques to address participants’ ambivalence about the program (Mayhew et al., 2020), whereas the “meet and greet” session in HWB was more informal, centered on boosting social connection, and answering participants’ questions. Thus, future research focused on understanding the mechanisms by which orientation sessions support engagement, and consequently, retention and adherence would be valuable. Importantly, when adaptations involved adding new elements, these were designed to be low-burden and integrated into existing program infrastructure, without substantially reducing feasibility.

This study has important limitations. Although our recruitment procedures were designed to maximize outreach to diverse racial and ethnic groups in this rural region, the perspectives represented across study phases were nonetheless limited. Notably, interviews were conducted only with mothers; the interview sample included only one individual whose preferred language was Spanish; adolescents were not included as CT or CAB members; and no pilot program participants identified as Native American. Because the goal of this project was a preliminary evaluation of the adaptation process, we focused on qualitative and quantitative indicators of feasibility and acceptability and did not examine whether adaptations improved clinical outcomes. Future randomized controlled trials comparing the adapted program to the base program are warranted. There have been calls for more systematic and detailed documentation of the processes by which interventions are adapted for local contexts (Sanders Thompson et al., 2015). Although the current co-creation process was unique to the specific communities in which the program was delivered, detailed reporting of this process and outcomes may support translation to other settings. It is possible that other less intensive lifestyle programs or alternative approaches to IHBLT may be more culturally acceptable or pragmatic for rural communities, suggesting that alternatives merit consideration. Study strengths include the use of multiple data collection and analytic methods, triangulation of qualitative data sources, and member reflections to enhance trustworthiness. The extended and intensive community engagement through the CT, CAB, and iterative adaptation process also represent strengths.

In conclusion, prolonged community engagement and multi-interest holder data collection resulted in an initial set of adaptations that supported acceptability of HWB for rural families with adolescents. Iterative rounds of focus group feedback and adaptation further improved feasibility and acceptability. Use of the FRAME-IS enabled systematic decision-making around adaptations while balancing fidelity to program components with flexibility to local needs. Our findings suggest that adapting IHBLT for local contexts can enhance acceptability, and that researchers and practitioners may benefit from extensive community engagement, multiple data sources, and iterative adaptation in collaboration with program developers, community members, and program participants.

## Supplementary Information

Below is the link to the electronic supplementary material.

ESM 1 (DOCX 26.4 KB)

ESM 2 (DOCX 51.9 KB)

ESM 3 (DOCX 14.9 KB)

## Data Availability

Data are available by request from the first author.
