# Supplementary material for: Health Without Barriers: Community-Engaged Adaptation of a Whole Family-Inclusive Intensive Health Behavior and Lifestyle Program in Rural Colorado
Source: Prev Sci. 2026 Jun 26;27(6):983–98. doi: 10.1007/s11121-026-01942-y (PMC13433621; doi:10.1007/s11121-026-01942-y)
Supplement: Supplementary file 1 [file 11121_2026_1942_MOESM1_ESM.docx]

| **Supplemental Table 1**. Program Attendance and Acceptability Ratings by Cohort | | | | | | | | | | |
| --- | --- | --- | --- | --- | --- | --- | --- | --- | --- | --- |
|  | **Cohort 1** | | **Cohort 2** | | **Cohort 3** | | **Cohort 4** | | **Cohort 5** | |
|  | **Attendance** | | | | | | | | | |
|  | Teen | Parent | Teen | Parent | Teen | Parent | Teen | Parent | Teen | Parent |
| Total Enrolled Participants^a^ | 5 | 3 | 5 | 4 | 6 | 5 | 4 | 3 | 9 | 7 |
| # of sessions attended: | N (%) |  |  |  |  |  |  |  |  |  |
| 1 to 4 | 2 (40) | 1 (33) | 1 (20) | 1 (25) | 2 (33) | - | - | - | - | 1 (14) |
| 5 to 7 | - | - | - | - | 1 (17) | 2 (40) | 2 (50) | 2 (67) | - | 1 (14) |
| 8 to 10 | 1 (20) | - | 4 (80) | 2 (50) | 3 (50) | 2 (40) | 2 (50) | 1 (33) | 4 (44) | 3 (43) |
| 11 or 12 | 2 (40) | 2 (66) | - | 1 (25) | - | 1 (20) | - | - | 5 (55) | 2 (29) |
| Median (IQR) | 10.5 (6) | 6^b^ | 9 (9) | 10.5^b^ | 7.5 (3) | 9.5 (4) | 7.5 (1.5) | 7^b^ | 11(1) | 10 (6) |
|  |  |  |  |  |  |  |  |  |  |  |
|  | **Acceptability** | | | | | | | | | |
|  | Teen | Parent | Teen | Parent | Teen | Parent | Teen | Parent | Teen | Parent |
| Total Respondents (% Enrolled) | 0^c^ | 0^c^ | 2 (40) | 4 (100) | 4 (67) | 3 (60) | 2 (50) | 3 (100) | 7 (78) | 3 (43) |
| **Would you participate in the program again?** |  |  |  |  |  |  |  |  |  |  |
| Definitely or Probably |  |  | 0 | 3 | 4 | 3 | 0 | 2 | 3 | 3 |
| Maybe |  |  | 0 | 0 | 0 | 0 | 2 | 1 | 2 | 0 |
| Probably Not or Definitely Not |  |  | 2 | 1 | 0 | 0 | 0 | 0 | 2 | 0 |
| **How much did you enjoy participating?** |  |  |  |  |  |  |  |  |  |  |
| Definitely or Mostly Enjoyed |  |  | 0 | 3 | 3 | 2 | 1 | 2 | 4 | 2 |
| Somewhat Enjoyed |  |  | 0 | 0 | 1 | 1 | 1 | 1 | 3 | 1 |
| Only Slightly or Did Not Enjoy |  |  | 2 | 1 | 0 | 0 | 0 | 0 | 0 | 0 |
| **Would you recommend the program to family/friends?** |  |  |  |  |  |  |  |  |  |  |
| Definitely or Probably |  |  | 0 | 3 | 4 | 3 | 1 | 3 | 4 | 3 |
| Maybe |  |  | 0 | 0 | 0 | 0 | 1 | 0 | 2 | 0 |
| Probably Not or Definitely Not |  |  | 2 | 1 | 0 | 0 | 0 | 0 | 1 | 0 |

Note: ^a^ Number of participants enrolled who attended at least one program session. IQR: Interquartile Range. ^b^ 3 or fewer participants, so Interquartile Range could not be calculated. ^c^ Acceptability questionnaires were not administered to participants in the first cohort.

**Supplemental Table 2**. Excerpt from the master adaptation tracker, a modified version of the Framework for Reporting Adaptations and Modifications to Evidence-based Implementation Strategies (FRAME-IS)(Miller et al., 2021).

| **Program Component** | **WHEN did the modification occur?** | **WHERE did the idea for the modification originate?** | **WHEN was the modification implemented?** | **CATEGORY of modification** | **WHAT was modified?** | **WHAT is the nature of the modification?** | **WHAT was the primary goal?** |
| --- | --- | --- | --- | --- | --- | --- | --- |
| Parent Curriculum | Pre-implementation/ planning/pilot | Community Translation and/or Interviews | Cohort 1 | Intervention Content | Facilitator manual/procedures | Adding elements | Increase acceptability/ satisfaction |
| Parent Curriculum | Implementation | Facilitators | Cohort 2 | Intervention content | Facilitator manual/procedures | Reordering of modules/segments | Improve feasibility |
| Mindfulness Curriculum | Implementation | Participant Focus Groups | Cohort 2 | Implementation | Facilitator manual/procedures | Adding elements | Improve effectiveness/ outcomes |
| Cooking & Nutrition | Implementation | Participant Focus Groups | Cohort 3 | Implementation | Facilitator manual/procedures | Adding elements | Increase acceptability/ satisfaction |
| Physical Activity | Implementation | Facilitators | Cohort 2 | Materials | Participant workbook | Adding elements | Improve effectiveness/ outcomes |
| Facilitator Training | Pre-implementation/ planning/pilot | Community Translation and/or Interviews | Cohort 1 | Training | Training procedures | Refining/adjusting elements | Increase acceptability/ satisfaction |
| General Operating Procedures | Implementation | Participant Focus Groups | Cohort 2 | Implementation | General program procedures | Adding elements | Increase retention |

Note: Additional columns not shown here for space include: When was the modification suggested (date); Describe the original suggestion for the modification (in this field we would describe the data/input received, for example, a quote from a focus group or feedback from a Community Advisory Board member); Describe the modification (in this field we would describe in detail the modification).

| **Supplemental Table 3**. Number of Adaptations Made to the Program Components, by Cohort | | | | |  |  |
| --- | --- | --- | --- | --- | --- | --- |
|  | Before Cohort 1 | After Cohort 1 | After Cohort 2 | After Cohort 3 | After Cohort 4 | After Cohort 5 |
| Program Domain | N (% of all changes) | | | | | |
| Parent Curriculum | 25 (45%) | 11 (20%) | 13 (23%) | 4 (7%) | 2 (4%) | 0 |
| Mindfulness Curriculum | 9 (43%) | 6 (29%) | 1 (5%) | 0 | 5 (24%) | 0 |
| Physical Activity | 4 (22%) | 11 (61%) | 1 (5%) | 1 (5%) | 1 (5%) | 0 |
| Cooking & Nutrition | 0 | 0 | 4 (50%) | 3 (38%) | 1 (13%) | 0 |
| Facilitator Training | 3 (21%) | 0* | 0* | 0* | 0* | 11 (79%) |
| General Operating Procedures | 1 (3%) | 5 (17%) | 7 (23%) | 6 (20%) | 10 (33%) | 1 (3%) |

*No new facilitators were trained after Cohorts 1, 2, 3, or 4.
