# Supplementary material for: Health Without Barriers: Community-Engaged Adaptation of a Whole Family-Inclusive Intensive Health Behavior and Lifestyle Program in Rural Colorado
Source: Prev Sci. 2026 Jun 26;27(6):983–98. doi: 10.1007/s11121-026-01942-y (PMC13433621; doi:10.1007/s11121-026-01942-y)
Supplement: Supplementary file 2 [file 11121_2026_1942_MOESM2_ESM.docx]

Healthy Living Program Southwest Post-Program Focus Groups: Parent Version

Grupos de sondeo posteriores al programa del programa de vida saludable en el suroeste: versión para

| **Question type** | **Sample questions** |
| --- | --- |
| Opening questions- not transcribed, builds rapport  Preguntas de apertura – no transcritas, construye buena relación | “Please tell us your name and preferred pronouns – if you have them and would like to share them… [insert ice breaker]”    Some icebreaker ideas:   - What’s the last movie you watched, and do you recommend it? - How many hours/days could you survive without a cell phone? - If you could invent a robot that could complete any daily task for you, what task would you program the robot to complete?   Por favor diga su nombre y pronombres preferidos, si los tiene y le gustaría compartirlos… [insertar rompehielos]”    Ideas de rompe hielo:   - ¿Cuál es la última película que viste? ¿La recomendarías? - ¿Si fueras un animal, cual serias y por qué?   Cuantas horas/días podrías sobrevivir sin tu teléfono? |
| Introductory questions- putting the group in the mindset of the topic  Preguntas de introducción – poniendo al grupo en la mentalidad del tema. | “What’s your best memory from the Healthy Living Program? What made this a positive moment for you?”  “Cuál es el mayor recuerdo que tienes del programa Vida Saludable? ¿Que hizo este un momento positivo para ustedes?”  What did you expect when you first started the program? What did you think you’d get out of it? In what ways did it meet or not meet your expectations?  ¿Qué esperabas cuando comenzaste el programa por primera vez? ¿Qué pensaste que te llevarías de él? ¿De qué maneras alcanzo o no alcanzo tus expectativas? |
| Healthy Living Education Component: Key questions- choose 1-2 questions from each section  Componente del programa La Vida Saludable: Preguntas claves- elija 1-2 preguntas de esta sección. | **1) Enabling Mechanisms** / Mecanismos habilitadores    “What was most helpful about the healthy living education part of the program?  ¿Qué fue lo más útil de la educación para vivir una vida salud del programa?      “What kept you engaged in that part of the program each week? What would you change to make the program more engaging?”  “¿Cuáles cosas los mantuvo involucrados en esa parte del programa cada semana? ¿Qué cambiaría para que el programa fuera más atractivo?”  What do you think about the amount of content that is being covered in class?   - Probe: What would you like to spend more time on? What would you like to spend less time on? One of the most important components of HeLP is goal setting. How did you like the goal setting component of the program? We’re interested in your thoughts on the process, the materials, and any other thoughts you have on this.   ¿Qué piensas sobre la cantidad de contenido que se cubre en clase?  - Sondeo: ¿A qué gustaría dedicar más tiempo? ¿En qué le gustaría dedicar menos tiempo? Uno de los componentes más importantes de HeLP es el establecimiento de objetivos. ¿Qué les pareció el componente de establecimiento de metas del programa? Estamos interesados en sus pensamientos sobre el proceso, los materiales y cualquier otro pensamiento que tenga sobre esto.  **2)**  **Home practice/ Practica en casa**    “I know you were encouraged to do some activities that you talked about during the group at home, like physical activity and making changes to your family’s routines. What’s it like to bring what you learned in the group home and try it out? What got in the way?  “Sé que los animaron a hacer algunas actividades hablaron de durante el grupo en casa, como actividad física y hacer cambios en las rutinas de tu familia. ¿Cómo fue traer lo que ustedes aprendieron en el grupo y incorporarlo en sus vidas diarias? ¿Cuáles fueron algunos desafíos para incorporal las lecciones en su vida?  What did you think of the resources and communication related to teen’s at-home practice? What might make this more helpful?  ¿Qué pensó de los recursos y la comunicación relacionados con la práctica en el hogar de los adolescentes? ¿Qué podría hacer esto más útil?    **3) Negative Aspects of Interventions/ asepctos negativos de la intervencion**  “What was one thing that was hard, upsetting, or annoying about the healthy living education part of the program? It’s okay to be honest with us! We want to make this the best program possible in the future.  Was there anything else that was hard, upsetting, or annoying?  “¿Qué fue algo que fue difícil, molesto o fastidioso sobre la parte del de la educación de salud del programa? ¡Está bien ser honesto con nosotros! Queremos hacer de este el mejor programa posible en el futuro.  ¿Hubo algo más que fue duro, molesto o fastidioso?  “Now… Imagine you have magical powers that can fix any problem at all instantly. Please tell us your magical solution to your main problem with the healthy living education part of the program?”  “Ahora… Imagínense que ustedes tienen poderes mágicos que pueden solucionar cualquier problema al instante. Por favor, díganos su solución mágica a su principal problema con la parte del programa de educación para una vida saludable.    **Or, if short on time...** o si hay poco tiempo    “What didn’t work for you with the healthy living education part of the program? What could help other parents and guardians to be more successful or enjoy that part of the program more?”  “¿Qué no funcionó para usted con la educación para vivir una vida más saludable del programa? ¿Qué podría ayudar a otros padres y tutores a tener más éxito o disfrutar más esta parte del programa?”  4) **Mindfulness/ atención** **plena**  Can you share your thoughts on the mindfulness portion of the program? What did you like about it or not like about it? What did you think about the mindful moment at the start of each session?  ¿Puedes compartir tus pensamientos sobre la parte de atención plena del programa? ¿Qué les gustó o no les gustó? ¿Qué pensaron sobre el momento consciente al comienzo de cada sesión? |
| Cooking Matters Component: Key questions- choose 1-2 questions from each section  Componente de Cooking Matters Preguntas claves- elija 1-2 preguntas de esta sección. | **1) Enabling Mechanisms/** mecanismos de capacitación    “What was most helpful about the Cooking Matters part of the program?  “¿Qué fue lo más útil de la parte del programa Cooking Matters?    “What kept you engaged in the Cooking Matters part each week?”  “¿Qué los mantuvo involucrados en la parte de Cooking Matters cada semana?”    **2) Negative Aspects of Interventions**  “What was one thing that was hard or annoying about the Cooking Matters part of the program? It’s okay to be honest with us! We want to make this the best program possible in the future.  Was there anything else that was hard or annoying?  “¿Qué fue difícil o molesto sobre la parte del programa Cooking Matters? ¡Está bien ser honesto con nosotros! Queremos hacer de este el mejor programa posible en el futuro.  ¿Hubo algo más que fue difícil o fastidioso?  **Or, if short on time...**    “What didn’t work for you with the Cooking Matters part of the program? What could help other families to be more successful or enjoy the Cooking Matters part of the program more?”  “¿Qué no funcionó para usted con la parte del programa Cooking Matters? ¿Qué podría ayudar a otras familias a tener más éxito o a disfrutar más de la parte del programa Cooking Matters?”. |
| General Questions- choose 1-2 from each subsection  Preguntas general- elije 1-2 preguntas de esta subsección | **Health Assessments/** Evaluaciones de salud  We are considering making changes to the information that is provided to you and your family after your teen does their health assessments. As a reminder the health assessments are the things like the finger stick, height and weight, and wearing the glucose or blood sugar monitor.  What type of information would you like to receive about the results of your teen’s health assessments?  Is there a particular way you’d like to receive that information? (For example, a phone call from a physician or an information sheet, or something else?)  Estamos considerando hacer cambios a la información que se le proporciona a usted y a su familia después de que su adolescente hace sus evaluaciones de salud (evaluaciones (punción en el dedo, altura, peso, etc). ¿Qué tipo de información le gustaría recibir sobre los resultados de las evaluaciones de salud de su hijo/a/ adolescente? ¿Hay alguna forma en particular en que le gustaría recibir esa información, por ejemplo, una llamada telefónica de un médico o una hoja informativa, o alguna otra cosa?  **Group Leaders/Group Cohesion**  “Some groups just ‘click’, and some groups take a while to build relationships, and some groups just don’t get along at all! Tell me a little about how your group got along. What factors are important for a group to create a sense of connection in a program like this?    “¡Algunos grupos simplemente ‘hacen clic’, y algunos grupos tardan un tiempo en construir relaciones, y algunos grupos simplemente no se llevan bien! Cuénteme un poco sobre cómo se llevaba su grupo. ¿Qué factores son importantes para que un grupo cree un sentido de conexión en un programa como este?  “We’re going to be hiring some leaders for future groups. What are some characteristics we should make sure our future group leaders have? How were your group leaders in those areas?”  “Vamos a contratar algunos líderes para futuros grupos. ¿Cuáles son algunas de las características que debemos asegurarnos de que tengan nuestros futuros líderes de grupo? ¿Cómo fueron los líderes de su grupo en esas áreas?”  **Process of Change/Maintenance**  This program was about living a healthier lifestyle within your family. Some of the things you learned might have been new to you or big changes. If you made any changes, what was that process like? If you could draw the shape of that process, would it be a straight path? A winding and bumpy road? A path with loops back to the beginning?  Este programa se trata de vivir un estilo de vida más saludable con su familia. Algunas de las cosas que aprendiste pueden haber sido nuevas para ti o de grandes cambios. Si hiciste algún cambio, ¿cómo fue ese proceso? Si pudieras dibujar la forma de ese proceso, ¿sería un camino recto? ¿Un camino curvas y lleno de baches? ¿Un camino con rampas para volver al punto de partida?  Now that the group is over, what are you doing to feel healthy and happy? What are you doing to support your child or children in being healthier and happier? What challenges are you encountering? What would help you get past those challenges?    Ahora que el grupo ha terminado, ¿qué estás haciendo para sentirte saludable y feliz? ¿Qué está haciendo para ayudar a su hijo o hijos a ser más saludables y felices? ¿Qué desafíos estás encontrando? ¿Qué te ayudaría a superar esos desafíos?  For future groups, we are thinking about ways that we can use technology to improve the program. What ideas do you have for ways that technology could be used to make the program more helpful to you and your family? This could be using technology to help with communication between program staff and participating families, for participating families to be in touch with each other, online resources, or other ideas!  Because setting and tracking goals is such an important part of the program, we are considering adding a technology component to support participants with tracking goals. What ideas do you have about technology tools that would be helpful for tracking goals in the program?   - Probe: What do you think about things like, a virtual goal calendar, reminders via text, or virtual goal journals?   Para los grupos futuros, estamos pensando en formas en que podemos usar la tecnología para mejorar el programa. ¿Qué ideas tiene sobre las formas en que la tecnología podría usarse para hacer que el programa sea más útil para usted y su familia? ¡Esto podría ser usar tecnología para ayudar con la comunicación entre el personal del programa y las familias participantes, para que las familias participantes estén en contacto entre sí, recursos en línea u otras ideas!    Debido a que el establecimiento y seguimiento de objetivos es una parte tan importante del programa, estamos considerando agregar un componente tecnológico para ayudar a los participantes con el seguimiento de objetivos. ¿Qué ideas tiene sobre las herramientas tecnológicas que serían útiles para el seguimiento de las metas del programa?  - Indague: ¿Qué piensa acerca de cosas como un calendario de objetivos virtual, recordatorios a través de mensajes de texto o diarios de objetivos virtuales?  **Multicultural Orientation**  Take a moment to think about what you would consider the three most important aspects of your identity – these could be things like your race or ethnicity, your gender, your employment, role as a parent or spouse, etc.  Tómese un momento para pensar en lo que consideraría los tres aspectos más importantes de su identidad; estos podrían ser cosas como su raza o etnia, su género, su empleo, su papel como padre o cónyuge, etc.  How did these identities influence your experience of the program? What were the things about the program that made you feel comfortable or uncomfortable expressing these identities? In what ways do you feel these identities were respected and supported within the program? Or in what ways were they not respected and supported?  ¿Cómo influyeron estas identidades en su experiencia del programa? ¿Cuáles fueron las cosas del programa que te hicieron sentir cómodo o incómodo al expresar estas identidades? ¿De qué manera siente que estas identidades fueron respetadas y apoyadas dentro del programa? ¿O de qué manera no fueron respetados y apoyados?  In what ways did you feel that your cultural background wasn’t respected and supported?  ¿De qué manera sintió que no se respetaba ni apoyaba su origen cultural?  How convenient and practical was it for you to attend the program? What would you change to make it more convenient and practical? You might consider things like location, time, frequency of meetings, or other things.  ¿Qué tan conveniente y práctico fue para usted asistir al programa? ¿Qué cambiarías para hacerlo más cómodo y práctico? Puede considerar cosas como la ubicación, la hora, la frecuencia de las reuniones u otras cosas. |
| Closing Questions – Choose 1-2  (must save some time for the final question in some form)  Preguntas de cierre- elije 1-2 preguntas (guarde tiempo para la pregunta final de alguna forma | “What was the single most important thing you learned in this group?”  “¿Qué fue lo más importante que aprendiste en este grupo?”    “Raise your hand if you would recommend this study to someone else. Tell me about why you raised your hand or why you did not.”  “Levanta la mano si recomendaras este estudio a otra persona. Cuéntame por qué levantaste la mano o por qué no lo hiciste”.  How valuable was this program to you on a scale of 1 to 5? How valuable do you think the program was for your child?  ¿Qué tan valioso fue este programa para usted en una escala del 1 al 5? ¿Qué tan valioso cree que fue el programa para su hijo?    “What did you learn about yourself by participating in this study/group?”  “¿Qué aprendiste sobre ustedes mismos al participar en este estudio/grupo?”  “What else do we need to know about your experience with the program?” [Some form of this question is required]  “¿Qué más necesitamos saber sobre su experiencia con el programa?” [Se require alguna forma de esta pregunta] |

Healthy Living Program Southwest Post-Program Focus Groups: Teen Version

Grupos de sondeo posteriores al programa Vida Saludable en el Suroeste: Versión para adolescentes

| **Question type** | **Sample questions** |
| --- | --- |
| Opening questions- not transcribed, builds rapport  Preguntas de apertura – no transcritas, construye buena relación | “Please tell us your name and preferred pronouns – if you have them and would like to share them… [insert ice breaker]”    Some icebreaker ideas:   - What’s the last movie you watched, and do you recommend it? - If you were an animal, what would you be and why? - How many hours/days could you survive without a cell phone?   Por favor diga su nombre y pronombres preferidos, si los tiene y le gustaría compartirlos… [inserte rompehielos]”    Ideas de rompe hielo:   - ¿Cuál es la última película que viste? ¿La recomendarías? - ¿Si fueras un animal, cual serias y por qué?   Cuantas horas/días podrías sobrevivir sin tu teléfono? |
| Introductory questions- putting the group in the mindset of the topic, optional  Preguntas de introducción – situar al grupo en la perspectiva del tema | “What’s your best memory from the Healthy Living Program? What made this a positive moment for you?”  “Cuál es el mayor recuerdo que tienes del programa Vida Saludable? ¿Que hizo este un momento positivo para ti?”  What did you expect when you first started the program? What did you think you’d get out of it? In what ways did it meet or not meet your expectations?  ¿Qué esperabas cuando comenzaste el programa por primera vez? ¿Qué pensaste que te llevarías de él? ¿De qué maneras alcanzo o no alcanzo tus expectativas? |
| Physical Activity Component: Key questions- choose 1-2 questions from each section  Componente de actividad fisica: Preguntas claves- elija 1-2 preguntas de esta sección. | **1) Enabling Mechanisms/** Mecanismos habilitadores    “What was most helpful about the physical activity part of the program?  “¿Qué fue lo más útil de la parte de actividad física del programa?    “What kept you engaged in the physical activity part each week?”  “¿Qué te mantuvo involucrado en la parte de la actividad física cada semana?”    **2)**  **Home practice / Practica independiente**    “I know you were encouraged to do some of the physical activities at home. What’s it like to practice these skills outside of the group? What helps you to practice on your own? What got in the way?    “Sé que te animaron a hacer algunas de las actividades físicas en casa. ¿Cómo es practicar estas habilidades fuera del grupo? ¿Qué te ayuda a practicar por tu cuenta? ¿Qué se interpuso en el camino?    **3) Negative Aspects of Interventions/** / **aspectos negativos de la intervencion**  “What was one thing that was hard or annoying about the physical activity part of the program? It’s okay to be honest with us! We want to make this the best program possible in the future.  Was there anything else that was hard, upsetting, or annoying?  “¿Qué fue algo que fue difícil o molesto sobre la parte de actividad física del programa? ¡Está bien ser honesto con nosotros! Queremos hacer de este el mejor programa posible en el futuro.  ¿Hubo algo más que fue duro, molesto o molesto?  “Now… Imagine you have magical powers that can fix any problem at all instantly. Please tell us your magical solution to your main problem with the physical activity part of the program?”  “Ahora… Imagina que tienes poderes mágicos que pueden solucionar cualquier problema al instante. Por favor, díganos su solución mágica a su problema principal con la parte de actividad física del programa.    **Or, if short on time..** o, si hay poco tiempo    “What didn’t work for you with the physical activity part of the program? What could help other teens to be more successful or enjoy the physical activity part of the program more?”  “¿Qué no funcionó para usted con la parte de actividad física del programa? ¿Qué podría ayudar a otros adolescentes a tener más éxito o a disfrutar más la parte de actividad física del programa?” |
| Mindfulness Component: Key questions- choose 1-2 questions from each section  Componente de atención plena: Preguntas claves- elija 1-2 preguntas de esta sección. | **1) Enabling Mechanisms/ mecanismos habilitadores**    “What was most helpful about the mindfulness part of the program?    “¿Qué fue lo más útil de la parte de la atención plena del programa?  “What kept you engaged in the mindfulness part each week?”    “¿Qué te mantuvo involucrado en la parte de la atención plena cada semana?”  **2) Facilitators/Barriers to Home Practice/ Facilitadores/barreras para la práctica en el hogar**    “I know you had some home practice from group. What’s it like to practice mindfulness on your own? What’s hard about practicing on your own? What helps you to practice more on your own?”  “Sé que tuviste algo de prácticas individuales ¿Cómo es practicar atención plena por tu cuenta? ¿Qué tiene de difícil practicar solo? ¿Qué te ayuda a practicar más por tu cuenta?    **3) Negative Aspects of Interventions**  “What was one thing that was hard or annoying about the mindfulness part of the program? It’s okay to be honest with us! We want to make this the best program possible in the future.  Was there anything else that was hard or annoying?  “¿Qué fue algo que fue difícil o molesto sobre la parte de la atención plena del programa? ¡Está bien ser honesto con nosotros! Queremos hacer de este el mejor programa posible en el futuro.  ¿Hubo algo más que fue difícil o molesto?  “Now… Imagine you have magical powers that can fix any problem at all instantly. Please tell us your magical solution to your main problem with the mindfulness part of the program?”  “Ahora… Imagina que tienes poderes mágicos que pueden solucionar cualquier problema al instante. Por favor, díganos su solución mágica a su problema principal con la parte de atención plena del programa.    **Or, if short on time...** o, si hay poco tiempo    “What didn’t work for you with the mindfulness part of the program? What could help other teens to be more successful or enjoy the mindfulness part of the program more?”  ¿Qué no funcionó para ti con la parte de la atención plena del programa? ¿Qué podría ayudar a otros adolescentes a tener más éxito o a disfrutar más de la parte de atención plena del programa? |
| Cooking Matters Component: Key questions- choose 1-2 questions from each section  Componente de Cooking Matters: Preguntas claves- elija 1-2 preguntas de esta sección. | **1) Enabling Mechanisms/ /mecanismos** de capacitación    “What was most helpful about the Cooking Matters part of the program?”  “¿Qué fue lo más útil de la parte del programa Cooking Matters?”      “What kept you engaged in the Cooking Matters part each week?”  “¿Qué te mantuvo involucrado en la parte de Cooking Matters cada semana?”    **2) Negative Aspects of Interventions/ aspectos negativos de la intervención**    “What was one thing that was hard or annoying about the Cooking Matters part of the program? It’s okay to be honest with us! We want to make this the best program possible in the future.  Was there anything else that was hard or annoying?  “¿Qué fue algo que fue difícil o molesto sobre la parte del programa Cooking Matters? ¡Está bien ser honesto con nosotros! Queremos hacer de este el mejor programa posible en el futuro.  ¿Hubo algo más que fue difícil o molesto?  **Or, if short on time..** o si hay poco tiempo    “What didn’t work for you with the Cooking Matters part of the program? What could help other teens to be more successful or enjoy the Cooking Matters part of the program more?”  “¿Qué no funcionó para ti con la parte del programa Cooking Matters? ¿Qué podría ayudar a otros adolescentes a tener más éxito o a disfrutar más de la parte del programa Cooking Matters?”. |
| General Questions- choose 1-2 from each subsection  Preguntas general- elije 1-2 preguntas de esta subsección | **Health Assessments**  If you chose to participate in the health assessments, what were you curious about after you completed those assessments? As a reminder, the health assessments were the things like the finger stick, height and weight, and wearing the glucose or blood sugar monitor. What information would you have liked to receive after your assessments?  Si eligió participar en las evaluaciones de salud, ¿sobre qué sintió curiosidad después de completar esas evaluaciones? Como recordatorio, las evaluaciones de salud incluyeron cosas como la punción en el dedo, la altura y el peso, y el uso del monitor de glucosa o azúcar en la sangre. ¿Qué información le hubiera gustado recibir después de sus evaluaciones?  **Group Leaders/Group Cohesion/ /** **líderes de grupo/Cohesión de grupo**  “Some groups just ‘click’, and some groups take a while to build relationships, and some groups just don’t get along at all! Tell me a little about how your group got along. What factors are important for a group to create a sense of connection in a program like this?  “¡Algunos grupos simplemente ‘hacen clic’, y algunos grupos tardan un tiempo en construir relaciones, y algunos grupos simplemente no se llevan bien! Cuénteme un poco sobre cómo se llevaba su grupo. ¿Qué factores son importantes para que un grupo cree un sentido de conexión en un programa como este?    “We’re going to be hiring some leaders for future groups. What are some characteristics we should make sure our future group leaders have or don’t have? How were your group leaders in those areas?”  “Vamos a contratar algunos líderes para futuros grupos. ¿Cuáles son algunas de las características que debemos asegurarnos de que nuestros futuros líderes de grupo tengan o no tengan? ¿Cómo fueron los líderes de su grupo en esas áreas?”  We’d like to create more opportunities for teens to act as leaders within the program. What ideas do you have for ways that we might do that? What type of leadership roles would you have liked to have during the program? If there were opportunities to be involved in the program in a leadership role now that you’ve completed the program, what types of things might you be interested in?  Nos gustaría crear más oportunidades para que los adolescentes actúen como líderes dentro del programa. ¿Qué ideas tienes sobre las formas en que podríamos hacer eso? ¿Qué tipo de roles de liderazgo le hubiera gustado tener durante el programa? Si hubiera oportunidades para participar en el programa en un rol de liderazgo ahora que ha completado el programa, ¿en qué tipo de cosas podría estar interesado?  **Maintenance**  “Now that the group is over, what are you doing to feel healthy and happy?” What challenges are you encountering? What would help you get past those challenges?  “Ahora que el grupo terminó, ¿qué estás haciendo para sentirte saludable y feliz?” ¿Qué desafíos estás encontrando? ¿Qué te ayudaría a superar esos desafíos?  **Multicultural orientation**  Take a moment to think about what you would consider the three most important aspects of your identity – these could be things like your race or ethnicity, your gender, being a student, being part of an after-school activity that you’re in, a sibling, etc.  Think about your experience in the program and how comfortable you felt expressing these parts of yourself. What were the things about the program that made you feel comfortable or uncomfortable expressing these parts of yourself? In what ways do you feel these identities were respected and supported within the program? Or in what ways were they not respected and supported? Do any specific moments stand out?  Tómese un momento para pensar en lo que consideraría los tres aspectos más importantes de su identidad; estos podrían ser cosas como su raza o etnia, su género, ser estudiante, ser parte de una actividad extracurricular en la que participa, un hermano, etc    Piensa en tu experiencia en el programa y en lo cómodo que te sentiste al expresar estas partes de ti mismo. ¿Cuáles fueron las cosas del programa que te hicieron sentir cómodo o incómodo al expresar estas partes de ti mismo? ¿De qué manera siente que estas identidades fueron respetadas y apoyadas dentro del programa? ¿O de qué manera no fueron respetados y apoyados? ¿Se destacan algunos momentos específicos?  **Mindful Moment**  What did you think about the mindful moment at the beginning of each session? In what ways was this helpful or not helpful?  Qué pensaste sobre el momento de atención plena al comienzo de cada sesión? ¿De qué manera fue útil o no útil?  **Technology**  We want to support participants in reaching their goals during and after the program. We’re considering how we can use technology to do that. What ideas do you have for ways that technology would have supported you during the program, or might support you now to continue what you learned in the program?  Queremos ayudar a los participantes a alcanzar sus metas durante y después del programa. Estamos considerando cómo podemos usar la tecnología para hacer eso. ¿Qué ideas tiene sobre las formas en que la tecnología lo habría apoyado durante el programa, o podría ayudarlo ahora a continuar con lo que aprendió en el programa? |
| Closing Questions – Choose 1-2  (must save some time for the final question in some form)  Preguntas de cierre- elije 1-2 preguntas (guarde tiempo para la pregunta final de alguna forma | “What was the single most important thing you learned in this group?”  ¿Qué fue lo más importante que aprendiste en este grupo?    “Raise your hand if you would recommend this program to someone else. Tell me about why you raised your hand or why you did not.”  “Levanta la mano si recomendaras este programa a otra persona. Cuéntame por qué levantaste la mano o por qué no lo hiciste”.    “What did you learn about yourself by participating in this study/group?”  “¿Qué aprendiste sobre ti mismo al participar en este estudio/grupo?”  “What else do we need to know about your experience with the program?” [Some form of this question is required]  “¿Qué más necesitamos saber sobre su experiencia con el programa?” [Se requiere alguna forma de esta pregunta] |

Healthy Living Program Southwest Post-Program Focus Groups: Facilitator Version

| **Question type** | **Sample questions** |
| --- | --- |
| Opening questions- not transcribed, builds rapport | “Please tell us your name and preferred pronouns – if you have them and would like to share them… [insert ice breaker]”    Some icebreaker ideas:   - What’s the last movie you watched, and do you recommend it? - If you were an animal, what would you be and why? - How many hours/days could you survive without a cell phone? |
| Introductory questions- putting the group in the mindset of the topic, optional | “What’s your best memory from the Healthy Living Program? What made this a positive moment for you?”  What did you expect when you first started the program? What did you think you’d get out of it? In what ways did it meet or not meet your expectations? |
| **Training and preparation** | Thinking back to the training you received before the program started…   - What were things that helped you feel prepared? - What do you wish you had received more training in before the program? - What were the major gaps in the training you received? - For future facilitators, what are the top 2 things it’s absolutely critical they know (or know how to do) before the program starts? |
| **Enjoyment and Challenges** | What were things you enjoyed about facilitating the program?  What were the things you enjoyed most about the program itself? Here, I’m talking about the content of the program, its philosophy, its format, etc.  What were things you didn’t enjoy about facilitating the program?  What were things you didn’t enjoy about the program itself?  What was the most rewarding part of facilitating? What changes could we make to the program so that involves more of what makes it most rewarding?  What was most challenging? Why was it challenging? What do you think could be done to make it less challenging?  What would you do differently if you were to facilitate another cohort, and why? |
| **Perceptions of participant experience** | Knowing that you all facilitated different parts of the program, what parts of the component you facilitated did you feel participants were most engaged with? This could be a specific session, a specific activity, or something more general.  What parts did you feel fell flat or weren’t as engaging? What would you add or change to make the program more engaging?  From your perspective, in what ways were participants benefitting the most from the program?  What seemed like the major barriers getting in the way of participants either enjoying or benefitting from the program? |
| **Multicultural orientation** | Did you feel you could be your authentic self in your role as a facilitator?  What aspect of the program felt misaligned or in conflict with any aspect of your identity or values?  Did you feel able to authentically connect to participants in the program?  In addition to being a facilitator, you are also, like the participants, a member of the La Plata County/Durango area community. You may also have had other things in common with participants in terms of background or identity. In what ways did this influence or shape your facilitation experience?  What strategies did you use to build and sustain rapport with participants? What was effective about these strategies? |
| Closing Questions – Choose 1-2  (must save some time for the final question in some form) | “What did you learn about yourself by facilitating this program?”  “What else do we need to know about your experience with the program?” [Some form of this question is required] |
