# Supplementary material for: Health Without Barriers: Community-Engaged Adaptation of a Whole Family-Inclusive Intensive Health Behavior and Lifestyle Program in Rural Colorado
Source: Prev Sci. 2026 Jun 26;27(6):983–98. doi: 10.1007/s11121-026-01942-y (PMC13433621; doi:10.1007/s11121-026-01942-y)
Supplement: Supplementary file 3 [file 11121_2026_1942_MOESM3_ESM.docx]

Adolescent Acceptability Questionnaire

1. If you could do it over, would you choose to participate in this program? (Definitely not, unlikely, maybe, probably, definitely yes)
2. How much did you enjoy participating in the program? (Not at all, slightly, somewhat, mostly, definitely)
3. Would you recommend this program to a friend? (Definitely not, unlikely, maybe, probably, definitely yes)

Caregiver Acceptability Questionnaire

1. If you could do it over, would you choose to participate in this program? Definitely not, unlikely, maybe, probably, definitely yes)
2. How much did you enjoy taking part in this program? (Not at all, slightly, somewhat, mostly, definitely)
3. Would you recommend this program to a friend and their family? (Definitely not, unlikely, maybe, probably, definitely yes)
